# A case report of Impella 5.5-induced accordion phenomenon of the right subclavian artery during coronary bypass surgery

**DOI:** 10.1093/ehjcr/ytag309

**Published:** 2026-05-05

**Authors:** Yuta Matsui, Takanari Fujita, Yousuke Sugita, Junichi Tazaki, Hisashi Sakaguchi

**Affiliations:** Department of Cardiovascular Medicine, Japanese Red Cross Wakayama Medical Center, 4-20 Komatsubara-dori, Wakayama 640-8558, Japan; Department of Cardiovascular Medicine, Japanese Red Cross Wakayama Medical Center, 4-20 Komatsubara-dori, Wakayama 640-8558, Japan; Department of Cardiovascular Surgery, Japanese Red Cross Wakayama Medical Center, 4-20 Komatsubara-dori, Wakayama 640-8558, Japan; Department of Cardiovascular Medicine, Japanese Red Cross Wakayama Medical Center, 4-20 Komatsubara-dori, Wakayama 640-8558, Japan; Department of Cardiovascular Surgery, Japanese Red Cross Wakayama Medical Center, 4-20 Komatsubara-dori, Wakayama 640-8558, Japan

**Keywords:** Case report, Coronary artery bypass grafting, Mechanical circulatory support, Impella 5.5, Accordion phenomenon

## Abstract

**Background:**

The Impella 5.5 has recently been introduced as a high-flow mechanical circulatory support device and is increasingly used to stabilize high-risk patients undergoing coronary artery bypass grafting (CABG). Although its peri-operative benefits have been described, reports on vascular complications remain limited.

**Case summary:**

A 73-year-old man on maintenance haemodialysis with progressive left ventricular dysfunction was scheduled for CABG under Impella 5.5 support. Pre-operative computed tomography (CT) showed a minimum luminal diameter of 7 mm in the right axillary artery, and the device was inserted through a prosthetic graft the day before surgery. Immediately after device placement, right radial pulsatility markedly decreased. Angiography revealed faint distal flow and an accordion-like deformation of the right subclavian artery. Because significant vascular injury was considered unlikely and the hand remained well perfused, CABG was performed without vascular reconstruction. After successful CABG, the Impella was removed on post-operative Day 1, resulting in immediate restoration of right radial pressure. Post-operative CT confirmed the absence of vascular injury, and the patient recovered without any neurological or ischaemic sequelae.

**Discussion:**

This case represents a rare instance of transient upper limb hypoperfusion caused by an accordion phenomenon of the subclavian artery during Impella 5.5 support. The phenomenon mimicked structural injury but resolved completely after device removal. The case underscores the importance of detailed pre-procedural vascular imaging, continuous limb perfusion monitoring, and awareness of reversible pseudo-lesions induced by Impella catheter-related straightening of tortuous vessels.

Learning points

- Axillary Impella 5.5 insertion can cause reversible flow reduction due to an accordion phenomenon, a pseudo-lesion that can mimic arterial dissection.
- Reduced flow at the Impella 5.5 implantation site after straightening of a tortuous artery should raise suspicion for an accordion phenomenon.
- Even with flow reduction due to an accordion phenomenon, Impella 5.5 support can be continued for the minimal required period as long as distal perfusion is preserved.

## Introduction

Current guidelines recommend coronary artery bypass grafting (CABG) in patients with multivessel coronary artery disease and a left ventricular ejection fraction (LVEF) ≤35% to improve long-term survival.^1^ However, peri-operative risk remains high in patients with severely reduced LVEF.^2^ Although off-pump CABG (OPCAB) may be associated with lower early mortality compared with on-pump CABG (ONCAB) in patients with impaired left ventricular function, it is also associated with a higher incidence of incomplete revascularization.^3^ Recently, Impella 5.5 (Abiomed, Danvers, MA, USA) has been used to provide intra-operative circulatory support during high-risk OPCAB, thereby facilitating completion of planned revascularization.^4^ However, Impella 5.5–related complications remain a concern.^5^ We report a case of transient upper limb arterial flow reduction caused by an accordion phenomenon following axillary Impella 5.5 insertion during planned OPCAB.

## Summary figure

**Figure:**
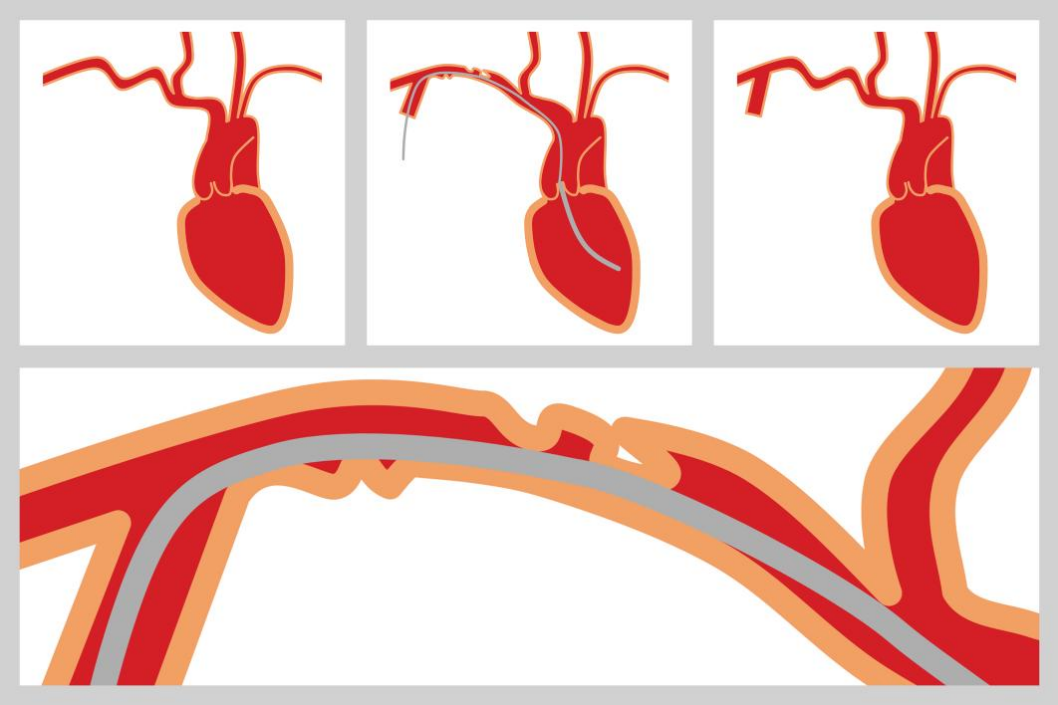
Schematic illustration of the proposed mechanism underlying transient right upper limb hypoperfusion. The upper left panel shows a tortuous right subclavian artery before Impella 5.5 insertion. The upper middle panel illustrates straightening of the vessel by the Impella catheter during insertion. The lower panel provides a magnified view demonstrating inward folding of the arterial wall with resulting luminal narrowing, consistent with the accordion phenomenon. The upper right panel shows resolution of arterial straightening and restoration of blood flow after Impella removal.

## Case presentation

A 73-year-old man undergoing maintenance haemodialysis had undergone percutaneous coronary intervention (PCI) of the proximal right coronary artery (RCA) 3 years earlier. At that time, moderate stenosis was present in the proximal left anterior descending coronary artery (LAD), but PCI was deferred because LVEF improved from 25% to 51% and he remained asymptomatic. Over the subsequent years, his LVEF gradually declined again to 28%, accompanied by increasing intolerance to haemodialysis, prompting admission for further evaluation. On admission, he had no chest pain or other ischaemic symptoms. Electrocardiography showed no ischaemic changes. Coronary angiography revealed total occlusion of the previously treated RCA with well-developed collateral flow from the left coronary artery and progression to severe stenosis of the proximal LAD.

**Figure 1:**
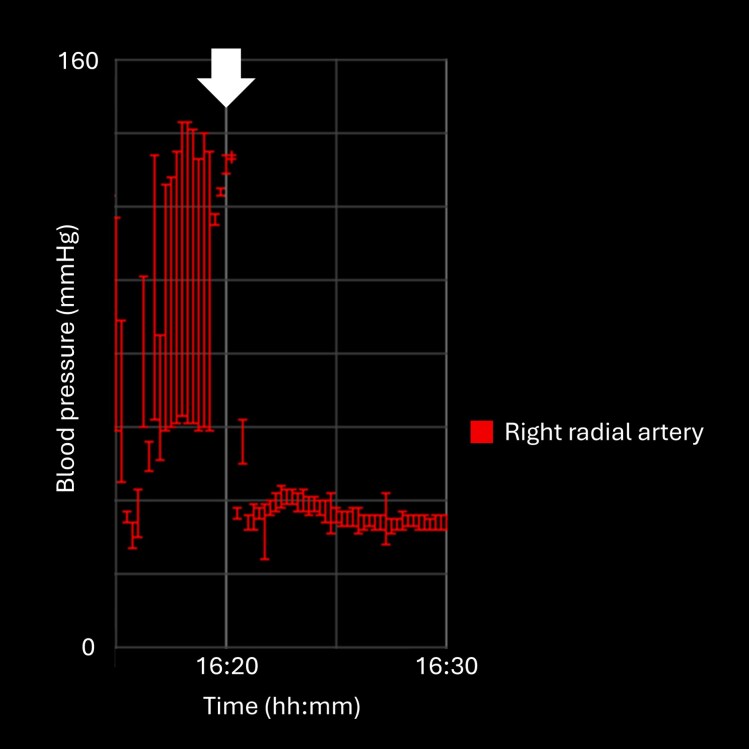
Trend chart of right radial arterial pressure showed an abrupt fall with near-complete loss of pulse pressure, resulting in systolic pressure decreasing to approximately 40 mmHg immediately after Impella 5.5 insertion at the time indicated by the white arrow.

**Figure 2:**
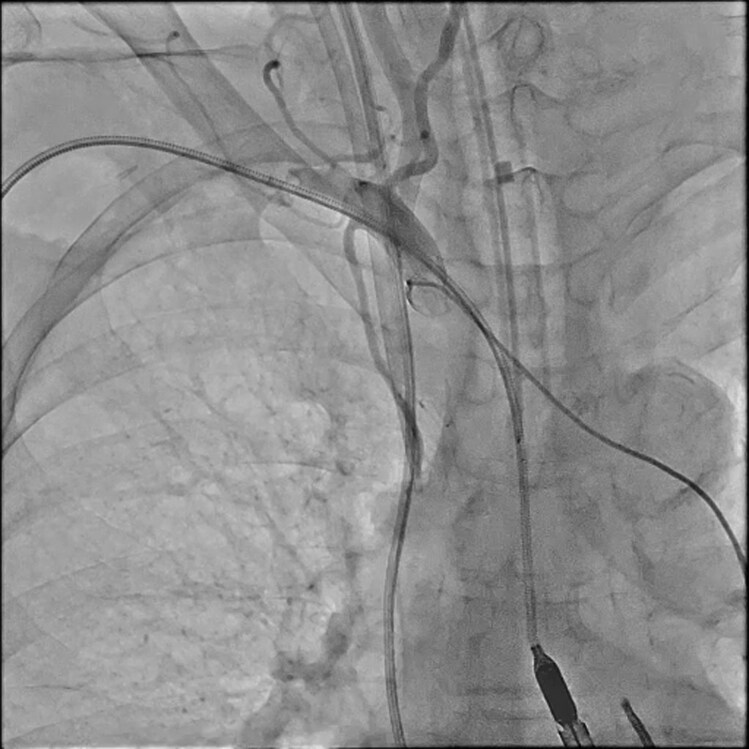
Angiography via a catheter positioned in the brachiocephalic artery, performed after a marked fall in right radial arterial pressure, demonstrated faint distal flow and an accordion-like deformation of the right subclavian artery immediately after Impella 5.5 insertion.

After a multidisciplinary heart team discussion, CABG was favoured over repeat PCI because in-stent occlusion of the previously treated RCA suggested limited durability of repeat PCI. Conventional ONCAB was considered high risk given severely depressed LVEF and dialysis dependency. However, OPCAB without mechanical support raised concern for intra-operative haemodynamic instability and incomplete revascularization. Intra-aortic balloon pump support was deemed insufficient due to limited augmentation. Therefore, OPCAB under Impella 5.5 support was planned using the left internal thoracic artery to the LAD and aortic saphenous vein grafts to the posterior descending and posterolateral branches. Contrast-enhanced computed tomography (CT) demonstrated a minimum luminal diameter of 7 mm in the right axillary artery. At our institution, Impella implantation is performed 1 day prior to surgery to allow early detection and management of insertion-related complications and to avoid prolongation of operative time. A 9-mm prosthetic graft was anastomosed to the right axillary artery, and the device was inserted through this graft. Although advancing the bulky pump housing of the Impella 5.5 from the axillary artery into the ascending aorta was somewhat challenging, the device was ultimately positioned successfully within the left ventricle. Immediately after device placement, the pulsatile right radial pressure disappeared, and the pressure decreased to approximately 40 mmHg (*Figure 1*). Subsequent angiography performed via the right femoral artery demonstrated faint distal flow and an accordion-like deformation of the right subclavian artery (*Figure 2*; Supplementary material online, *Video S1*). Although arterial dissection could not be completely excluded, vascular reconstruction was deferred given that the exposed adventitia appeared intact and distal perfusion remained clinically adequate. The sheath inserted into the right femoral artery for angiography was later utilized for arterial pressure monitoring.

**Figure 3:**
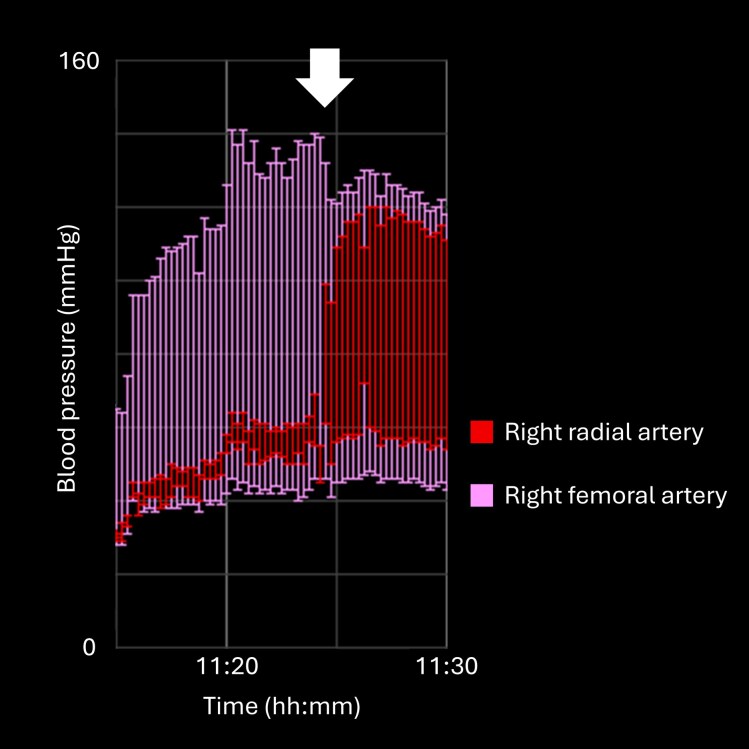
Trend chart of right radial arterial pressure showed immediate recovery of systolic and diastolic pressures and equalization with femoral arterial pressure after Impella 5.5 removal at the time indicated by the white arrow.

**Figure 4:**
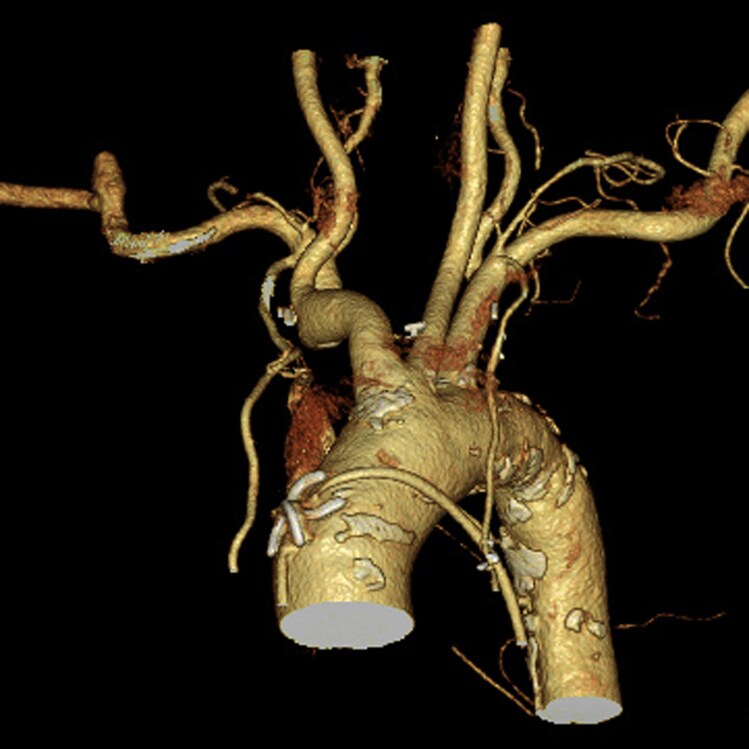
Post-operative computed tomography confirmed the absence of vascular injury.

The Impella 5.5 was set at performance level P8, providing approximately 4.3 L/min of flow. The CABG procedure was performed as planned. Proximal anastomoses of the saphenous vein grafts were performed using the Enclose II (Peters Surgical) proximal anastomosis assist device. Intra-operative haemodynamics remained stable with Impella support. The device was removed on post-operative Day 1, after which right radial pulsation immediately returned to baseline and equalled femoral pressure (*Figure 3*). Post-operative CT confirmed the absence of vascular injury (*Figure 4*). The patient developed post-operative pneumonia but improved with antibiotics and was extubated on post-operative Day 5. Haemodialysis was resumed uneventfully, and echocardiography before discharge showed improvement of the LVEF to 35%. He had no neurological or ischaemic sequelae in the right upper limb and was discharged home on post-operative Day 46. At 3-month follow-up, he remained clinically stable, continued maintenance haemodialysis without difficulty.

## Discussion

Vascular complications remain a notable concern during Impella 5.5 support. In a large cardiogenic shock registry including 972 patients treated with Impella 5.5, limb ischaemia, haematomas, and vascular events requiring surgical intervention were observed in 1.8%, 7.6%, and 1.4% of cases, respectively.^5^ These findings highlight the need for meticulous pre-procedural vascular assessment. The recent consensus statement on the clinical management of the Impella 5.5 recommends pre-procedural evaluation of the potential access sites using CT angiography or ultrasound to assess vessel size, tortuosity, calcification, and anatomic anomalies.^6^ In the present case, the axillary artery diameter was 7 mm and met the recommended minimum vessel size for axillary Impella 5.5 insertion.

In this case, angiographic findings were consistent with the accordion phenomenon, also referred to as the concertina effect or pseudo-lesion. This phenomenon arises when a tortuous artery is longitudinally stretched by a stiff device, causing transient inward folding of the vessel wall that mimics stenosis or dissection. First described during PCI,^7^ it has also been reported in other vascular territories, such as the carotid arteries.^8^ However, to our knowledge, no prior report has described this phenomenon resulting from an Impella catheter. Straightening of the tortuous right subclavian–brachiocephalic segment by the Impella catheter likely precipitated the finding.

Definitive diagnosis would require removal of the stretching device to confirm resolution. However, given the technical difficulty of insertion and preserved distal perfusion, device withdrawal solely for diagnostic confirmation was not pursued. Complete recovery of flow after Impella removal on post-operative Day 1 supported the diagnosis of an accordion phenomenon rather than fixed obstruction or dissection.

If distal upper limb perfusion had been more severely compromised due to an accordion phenomenon, temporary distal perfusion could have been attempted by inserting a sheath into the brachial artery and connecting it to the existing femoral arterial sheath to establish an extracorporeal bypass. Similar strategies have been reported in cases of limb ischaemia during femoral Impella support managed with contralateral femoral bypass.^9^ Alternatively, conversion to direct aortic insertion of the Impella 5.5 to avoid catheter traversal of the right subclavian–brachiocephalic segment, or conversion to conventional ONCAB, could have been considered.

This case highlights the importance of comprehensive vascular evaluation and continuous perfusion monitoring in patients receiving axillary Impella 5.5 support. Pre-operative imaging should be routinely performed to minimize risk. Intra-operative angiography can aid in detecting early flow compromise. In conclusion, while the Impella 5.5 provides highly effective circulatory support, clinicians must remain vigilant for reversible access-related flow disturbances such as the accordion phenomenon, which may mimic structural vascular injury but often resolves following device withdrawal.

## Supplementary Material

ytag309_Supplementary_Data

## Data Availability

The data underlying this article are available in the article and in its online Supplementary material.

## Lead author biography

Dr Yuta Matsui was born in Osaka, Japan, and graduated from the University of Miyazaki School of Medicine. After post-graduate training at a tertiary emergency and critical care centre, he is now a staff cardiologist at Japanese Red Cross Wakayama Medical Center. He provides comprehensive cardiovascular care, with a focus on patients with reduced left ventricular systolic function, coronary revascularization, cardioprotective pharmacotherapy, and cardiac rehabilitation. Influenced by his background, he also has a strong interest in primary prevention and community-based cardiovascular care.

## Notes

**Consent:** Written informed consent for submission and publication of this case report has been obtained from the patient in line with COPE guidance.
